# Monotonic loading performance of GFRP beam-column joints connected with slotted–hole bolts

**DOI:** 10.1371/journal.pone.0272136

**Published:** 2022-07-27

**Authors:** Xiao Xiao, Lei Xie, Yan Wang, Naxin Dai, Xiangdong Yin

**Affiliations:** 1 School of Civil Engineering, University of South China, Hengyang, Hunan Province, China; 2 Centre for Future Materials, University of Southern Queensland,Toowoomba, Australia; University of Vigo, SPAIN

## Abstract

Nowadays there are many types of glass fiber reinforced polymer(GFRP) composite beam and column joints, such as standard connection, bolted through connection, angle steel connection, tube connection and so on, most of which connected by high-strength bolts with round holes. In this paper, monotonic loading tests on GFRPcomposite beam and column joints connected by slotted-hole bolts were conducted. To compare the performance of different joints, two groups of specimens were used in this study; one of group was the beam-column joints connected by the angle steel, and other group was connected by the tube connection. Specimens with different bolt holes, side plate reinforcement condition, and different bolt pre-tightening forces were studied. Failure modes, bending moment curves, plastic rotation, and yield stiffness of the two groups of joints were compared. Results showed thatthe ultimate bending moment bearing capacity of specimens with side plates could be increased by 30%. Under the same conditions, the bearing capacity of the tube joints was about 10% larger than that of the angle steel joints. Although the bearing capacity of joints was not increased by using slotted holes, plastic rotation capacity and yield stiffness of joints with slotted-hole bolts were 1.1 times than that of the ordinary round-hole bolts joints.

## Introduction

There are mainly two kind of fiber reinforced polymer (FRP) composite materials are increasingly applied in civil engineering application in recent decades, namly CFRP (carbon fiber reinforced polymer) and GFRP (glass fiber reinforced polymer).

CFRP composite materials are characterized by their high Yong’s modulus, low volume desity and low coefficient of thermal expansion, but carbon fibers of CFRP result from the graphitization of organic rayon or polyacrylonitrile (PAN) textile fibers under an inert atmosphere at more than 2,000°C. During the process of graphitization, fibers are put under tensile stress: the greater the stress exerted, the higher the Young’s modulus obtained [1]. Besides, the increase of modulus is counterbalanced by a reduction of strength.

Although CFRP composite materials have higher elastic modulus and tensile strength than GFRP composites, the application in civil engineering are limited due to the high cost [2].

GFRP composite materials have gained popularity in replacing CFRP in civil engineering by characteristic of their light weigh, high strength, corrosion resistance and low maintenance [3–6].

GFRP composite materials consist of high-strength fibres embedded in a thermoset resin matrix. Glass fibers are mostly produced in the standard type of E-glass, S-glass, C-glass, D-glass, and L-glass. E-glass fibres in polyester or vinylester resin-based matrix are most commonly used in structural engineering application [1,2].

All glass types have a very high strength-weight ratio, although glass fibers are among the synthetic inorganic fibers with the highest density. Besides this, the following are the advantages offered by glass fibers and particularly by E-glass compared to other materials [1,2]:

- Ratio between high tensile strength and high resistance: with the equivalent weight of glass, the fiber has twice the strength of a steel wire.
- Dimensional stability: glass does not shorten or lengthen with varying environmental conditions. Glass fibers show a maximum lengthening of 3% before failure.
- High thermal resistance: glass fibers show good performance in applications under high temperatures. They preserve 50% of their tensile strength at a temperature of 340°C.
- Good electrical properties: glass has a low dielectric constant and good insulation capacity.
- High fire resistance: glass does not burn or set fire.

Pultrusion is the cheapest way to produce standard FRP profiles for structural use. With the commercialization of pultrusion technology, pultruded GFRP profiles received more interestes as structural members in building and bridge construction [7–11].Standard pultruded GFRP profiles resemble their steel counterparts. Complex shapes can be assembled through high-strength bolting to produce GFRP structures [12,13].

Typical pultruded GFRP profiles consist of two E-glass reinforcements of continuous filament mats and unidirectional fibre rovings. The strength and stiffness in longitudinal direction is provided by the unidirectional rovings and in transverse direction by the continuous filament mat [1,2]. Tensile strength of pultruded GFRP material in the longitudinal direction is in the range of 200–300 MPa with modulus of elasticity of 20–30 GPa. The material properties in the transverse direction are about one third of its longitudinal value. The mass of GFRP shapes is 25% of steel counterparts [1,2,9–13].

Joints play an important role in a structural system, and their performance directly affects the stiffness, ductility, and bearing capacity of structural systems [14]. Joints connected by bolts and connectors (such as steel tube connection, angle steel connection, etc.) can withstand a certain amount of bending moment and a corresponding rotation. Many scholars have carried out a lot of research work on such joints and achieved good results [15–17]. Considering the low Young’s modulus of GFRP profiles of only 30 GPa, semi-rigid connections are needed to improve the whole structural stiffness [18,19]. Smith et al. [20] found the rigidity of tubular section increased by 25% and strength by 280% comparing to the I-shaped section and tubular section under the same bending stiffness. This is because the tubular section improves buckling characteristics of the web, torsional stiffness, and axial stiffness. Bank et al. [21] investigated the connection of I-beam and column of GFRP through bolts and angle steel. To reduce the separation of the area where the column web was connected to the beam, a piece of GFRP plate was used to reinforce the connection area of beam and column on the surface of the GFRP column. Quareshi and Mottram [22,23] studied the response of pultruded GFRP members with bolted steel web cleats and the moment-rotation response of nominally pinned joints in frames of pultruded shapes. The effect of number and location of steel bolts required in the connection of GFRP beam and column joints were investigated, and it is found that satisfactory mechanical properties could be obtained with only two bolts. Luo et al. [24], Wu et al. [25] formed tube connector by welding steel pipe to steel end plates and inserted outside of tube into GFRP beam through bolted connection, and investigated the joint and member capacities experimentally and numerically. Using the similar tube connector, Zhang et al. [26] studied GFRP beam-column joint through epoxy adhesive bonding method, and found thatthe joint with adapted end plate connection had good ductility and deformation. Liu et al. [27] conducted an experimental investigation of the load-bearing behavior and ductility of GFRP hybrid double-lap joints composed of both adhesively-bonded and bolted connection parts, and found that the hybrid joints with multidirectional adherends and flexible adhesive exhibited high joint efficiencies and excellent ductility and thus may increase the overall safety of redundant engineering structures composed of brittle FRP members. Zhang et al. [28] also reviewed the numerical analysis of hybrid (bonded/bolted) GFRP composite joints and summarized an up-to-date progress in FEM analysis for this kind of joints. Jawed Qureshi et al. [29] presented test results from eight full-scale pultruded FRP beam-to-column joint subassemblies. The connection method is bolting or ‘hybrid’ combining both bolting and bonding. It was found that using steel cleats instead of GFRP resulted in a 50% increase in the moment resistance.

From studies that have been reported on the performance of GFRP beam-column joints, it is found that limited improvement on the mechanical properties of joints by using connector (tube or angle connections) with bolting or combining both bolting and bonding [30–34]. To improve the deformation capacity of GFRP beam-column joints, the method of slotted holes was proposed and investigated in this paper. Monotonic loading on the beam-column joints were applied to study the seismic performance of joints. Mechanical performance including failure modes, bearing capacity, plastic rotation, and yield stiffness of new joint were analysed comprehensively.

### Experimental investigation

#### Specimen design

**Fig 1:**
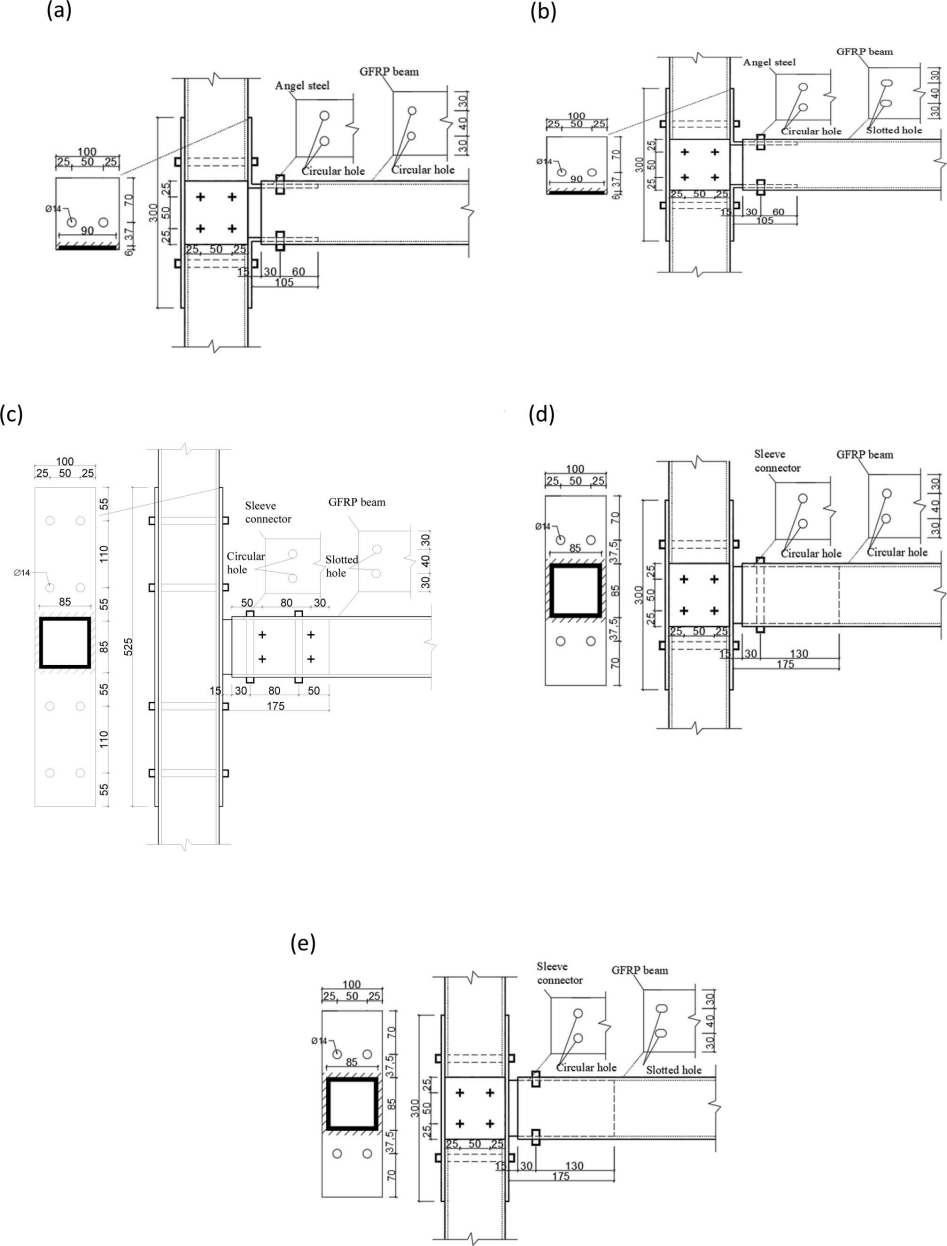
Details of specimens. (a) J1;(b)J2;(c)T1;(d)T2;(e)T3.

All specimens used pultruded box section of 100×100×5 mm made of GFRP composite material. The beam length was 1000 mm, and the column length was 1400 mm.GFRP beam and column joints with tubular sections were selected as the specimens in the test. The specimens were divided into two groups, namely, joints connected with angle steel (J1 and J2) and joints connected with tube (T1, T2, and T3). Fig 1 presents the configuration of speciemens.

**Fig 2:**
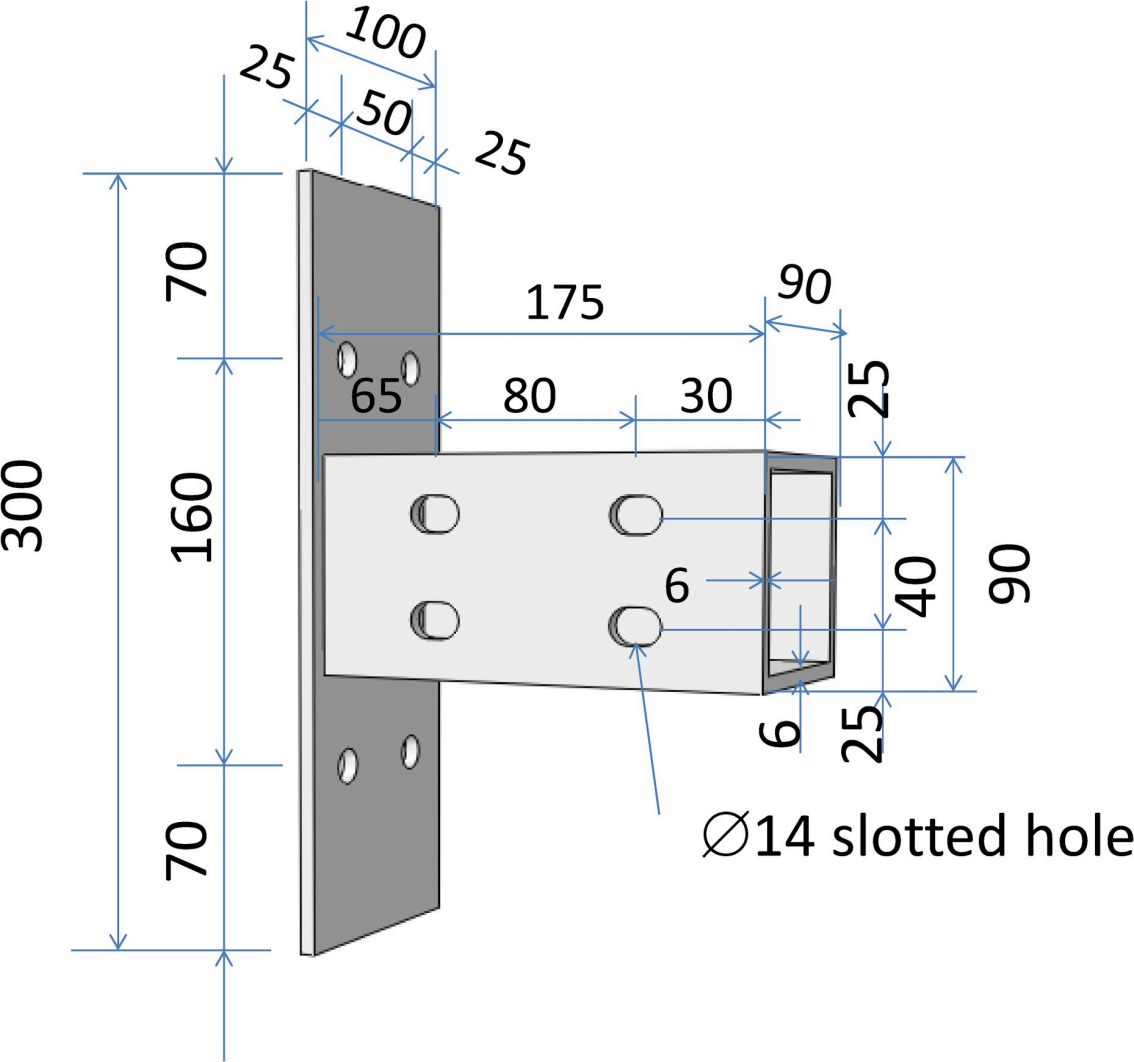
Details dimensions of tubular connectors(dimensions in mm).

As shown by Fig 1, the angle-steel joints (J1 and J2) connected the upper and lower flanges of GFRP beams to GFRP columns through angle steel and high-strength friction bolts. The thickness of side plate and endplate was 6mm and 4mm, respectively. For both of J1 and J2, the single-side bolts were adopted as high strength bolts on beam, and the pretension was 10kN. The difference between J1 and J2 was that bolt holes of J1 joints for angle steel branches of GFRP beam flanges were round holes, while J2 adopted slotted holes. The tube joints (T1, T2, and T3) inserted a tube into the GFRP beam and connected it with high-strength friction bolts. The steel tube was made of four steel plates with the size of 175×90×6 mm, as shown in Fig 2. The tube was welded to steel plates to form a connector. which was shown in Fig 2. GFRP column was connected to steel tube by endplates with high-strength through bolts, 8mm of thickness for T1 and 6mm for T2 and T3, respectively. GFRP beam was connected to steel tube directly by high-strength friction bolts. Through bolts used for T1 and T2, single-side bolts for T3. Amount of bolts for T1-T3 was 8, 2 and 4. Among them, bolt holes connected by GFRP flange of T2 joint and steel plates were round holes, while T1 and T3 joints were slotted holes. The diameter of bolt holes on the GFRP beam for T1, T3, J2 specimens was 14 mm and then slotted holes were drilled manually. The length of the slotted holes was 6 mm. For T2 and J1 joints, GFRP beams were ordinary round holes with a diameter of 14 mm. In order to avoid reduction of contact area between nuts and plates after slotted holes, an additional steel gasket was used between them.

#### Experimental material

All specimens used pultruded GFRP profiles consist of two E-glass reinforcements of continuous filament mats and unidirectional fibre rovings. where Young’s modulus of GFRP and steel is 32.2GPa and 203GPa, respectively; and Poisson’s ratio of GFRP and steel is 0.32 and 0.28, respectively.

Q235 grade steel was used for angle steel and tube, high strength friction bolts were used for beam web and flange connection, and a release torque wrench was used to apply pre-tightening force for the bolts. All bolts on the GFRP beams were 8.8-grade M12 high-strength friction hexagon bolts with an anti-slip coefficient of 0.45 and connected with hex nuts and washers of the same grade and diameter. The total length of the bolts was 14cm. All GFRP columns were made of 8.8-grade M12 high-strength through bolts. The details of the specimens were shown in Table 1. The parameters of Young’s modulus, Poisson’s ratio and compressive strength of various materials were measured by experiments, and the results were listed in Table 2.

**Table 1:** Specimen components.

Specimen	Slotted hole length (mm)	Thickness of side plate (mm)	Thickness of endplate (mm)	High strength bolts on beam		
				Amount	Bolt type	Pretension(kN)
**J1**	0	6	4	4	Single-side	10
**J2**	6	6	4	4	Single-side	10
**T1**	6	/	8	8	Through	15
**T2**	0	6	6	2	Through	5
**T3**	6	6	6	4	Single-side	5

Note: 1) The slotted hole length "0" represented the round hole.

2) In the bolt type, Through means that the bolt was connected through upper and lower flange of beams, and Single-side means that the bolt was connected through only one side flange of beams.

3) The bolts on columns were belong to high strength pressure bearing type, and the bolts on beams were belong to high strength friction type.

**Table 2:** Material parameters of specimens.

Material name	Properties	Value
GFRP	Young’s modulus *E*_G_ (GPa)	32.2
	Poisson’s ratio *μ*_*G*_	0.32
	Tensile Strength *f*_*u*_ (MPa)	307
	Shear strength *f*_*v*_ (MPa)	26.7
Angle steel, steel tube, side plate	Young’s modulus *E* (GPa)	203
	Poisson’s ratio *μ*	0.28
	Yield strength *f*_*y*1_ (MPa)	503
8.8 grade M12 High-strength bolts	Yield strength *f*_*y*2_ (MPa)	800

#### Test equipment and loading system

**Fig 3:**
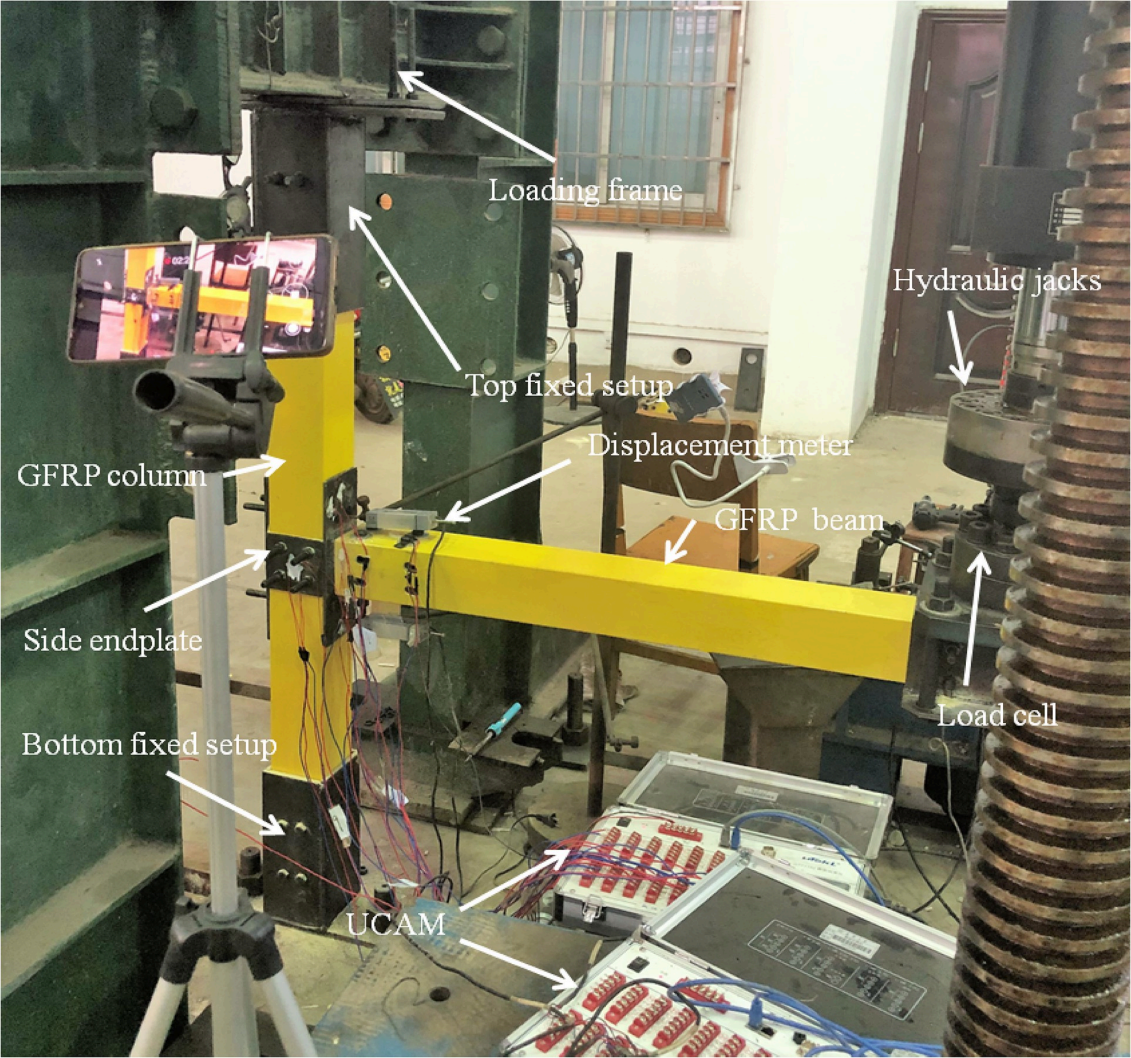
Experimental set-up for specimens.

The test loading device was a single channel electro-hydraulic servo loading system. The level of actuator deformation range was 200 mm, which could exert the maximum load of 250 kN. To facilitate loading, the GFRP column was fixed under the loading device. By applying a vertical displacement actuator in the beam end and connected actuator by a steel tube at GFRP beam loading point, it was to ensure the stability of loading position and keeping verticality of actuators. Solid wood blocks were inserted into GFRP beams to avoid local compression failure at loading points. Considering the gap between bolts and bolts holes during installation of specimens, a preloading test was carried out first. The loading device was shown in Fig 3. Displacement recorded by displacement meters set at different distances along GFRP beam from the joint to calculate the rotation angles of joints according to Eq (1):

$$\theta ={tan}^{-1}\frac{L_{2}-L_{1}}{L-0.3}\frac{180}{\pi }$$ (1)

Where *L* was the horizontal distance between loading point and column edge of joint, whose value was 950 mm; *L*_*1*_ and *L*_*2*_ were the vertical displacements measured by displacement meters installed at points of *CL*1 and *CL*2, respectively; The distance between the displacement meter of *CL1* and the contact end of GFRP beam and column joint was 0.3 m.

The bending moment of the joint was calculated according to Eq (2).

M=P·L (2)

Where *P* was the vertical force exerted on the loading point; *L* was the horizontal distance between the loading point and column edge, whose value was also 950 mm, as the same as Eq (1). As a result, the relation of moment-rotation of joints could be obtained conceptually.

**Fig 4:**
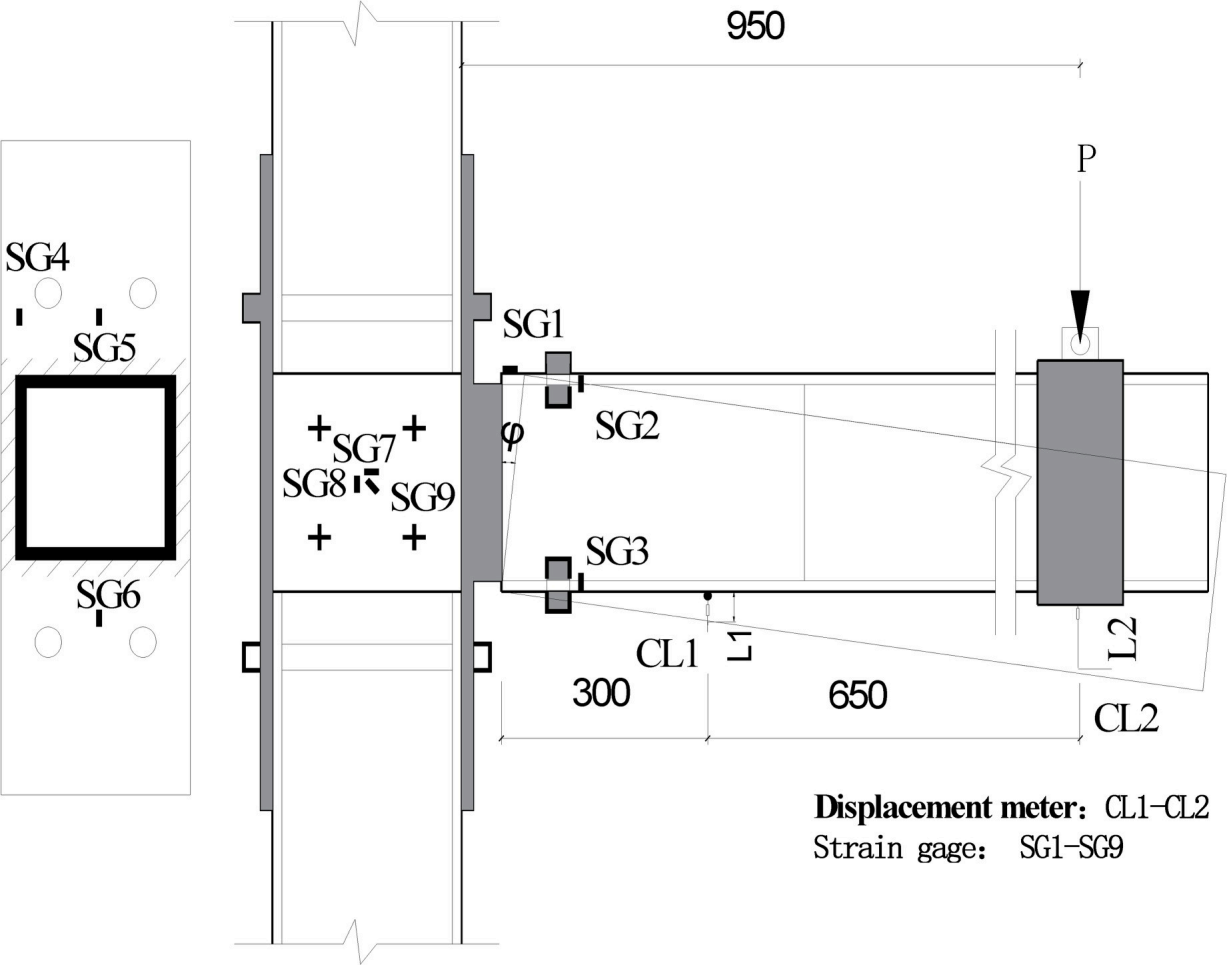
Diagram of strain gage and displacement meter.

In order to monitor the failure of beam-column joints, three strain gauges (SG1, SG2, and SG3) were installed on the surface of the GFRP beam to detect the shear force of the GFRP beam, to be specific, SG1 was installed on top of the GFRP beam, at the middle between two bolts, SG2 and SG3 were installed on side face of beam, closed to top and bottom surface of beam respectively, to detect the failure of the GFRP beam. Three strain gauges (SG4, SG5, and SG6) were installed on the end plate connected with steel tube, and strain gauges (SG7, SG8, and SG9) were installed on GFRP column to monitor shear stress at column joint area (as shown in Fig 4).

According to ASTM D1761 [35], displacement control was adopted during monotonic loading. Preloading was carried out first to eliminate the influence of gaps between bolts and component connection. Formal loading was carried out after displacement was back to zero, with a loading rate of 2 mm/ min.

### Experimental results

**Fig 5:**
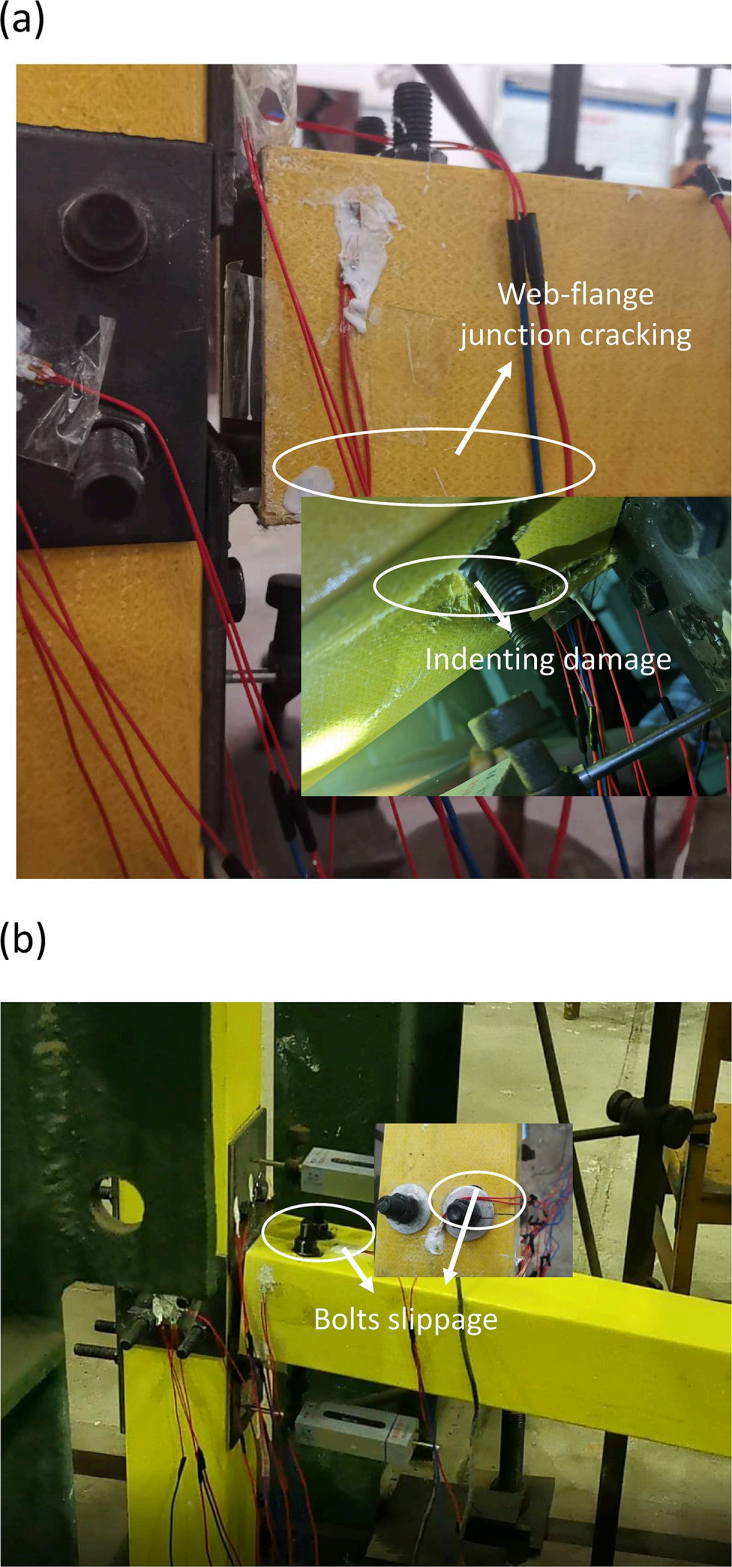
Failure modes of specimens of group J. (a) J1; (b) J2.

**Fig 6:**
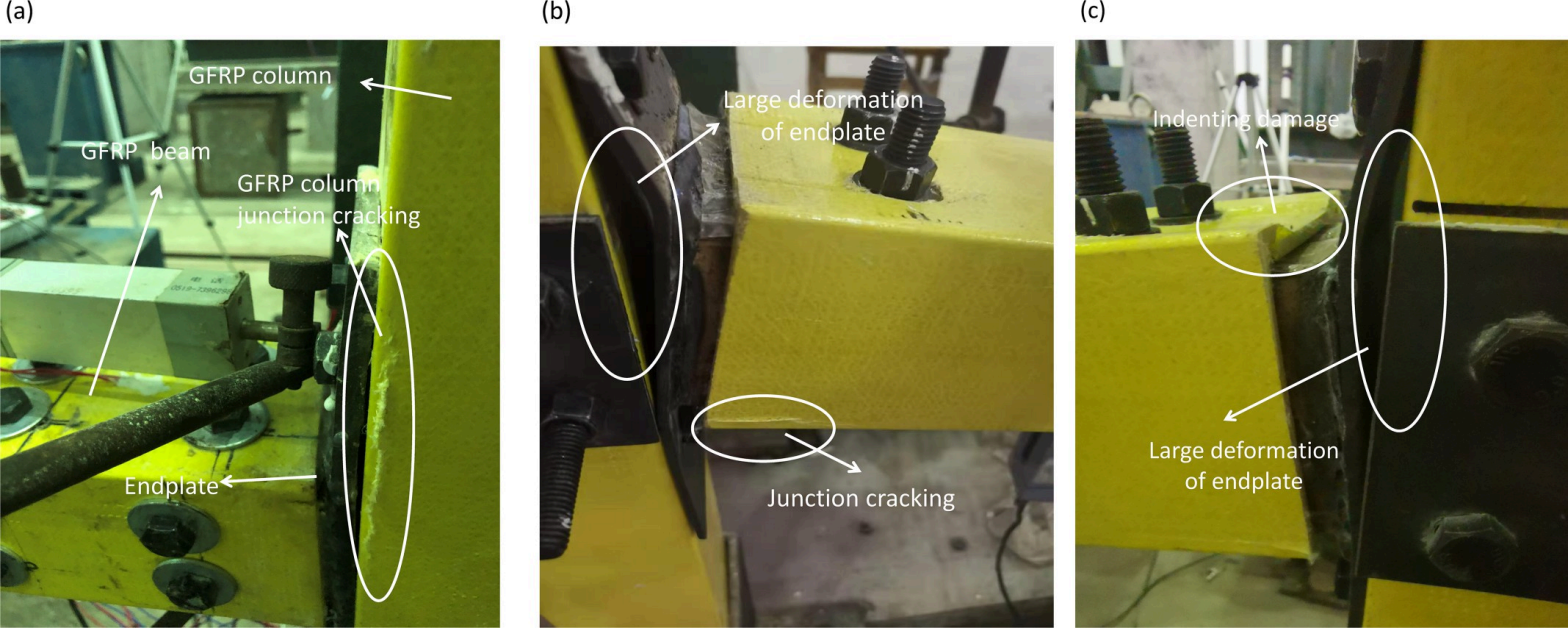
Failure modes of specimens of group T. (a) T1; (b) T2; (c) T3.

By recording the test process with a camera, the test results of five specimens in two groups of joints were obtained, and the detailed failure mechanism of each specimen was analyzed. Finally, the failure modes of group J were shown in Fig 5, and failure modes of group T were shown in Fig 6.

For J1 joint in group J, when the displacement of beam end was loaded to 30mm, slippage of bolts on the beam occurred. Because this joint has not been slotted-hole processed, when the displacement of the beam end reached 66 mm, cracks began to appear at the junction between the beam web and the flange because of "slip stage" for this joint was relatively short. When the displacement of beam end was loaded to 84 mm, the beam web was torn. At this moment, the joint could continue to resist loading. When displacement increased to 150 mm, the joint was completely crushed. The failure modes were shown in Fig 5(A).While for J2 joint, it was different for failure mode. When the displacement of beam end loaded to 28 mm, relative slip occurs between plates due to tensile stress of the component beyond the maximum contact friction between components, which located at GFRP bolt hole on the bearing pressure along the direction of GFRP beam flange. High-strength friction bolts of J2 joint began sliding phase because of the slotted hole on bolts for beam. Since the sliding stage of loading for the J2 joint was longer, friction sliding accompanied by sound, as shown in Fig 5(B). When the displacement raised to 86mm, GFRP material was torn and destroyed. The reason was that the upper end of angle steel was under tension and the lower end was under compression, so that squeezed the upper and lower flange of the beam. When the displacement of the beam end increased to 150mm, the GFRP beam crushed completely.

For T1 joint in group T, as shown in Fig 6(A), the joints cannot meet the requirements of strong columns and weak beams principle due to the absence of front and rear side plates on GFRP columns, so the failure mode of this joint was crack of GFRP column and upper flange of GFRP beams torn by tension.

For T2 joint, as shown in Fig 6(B), the failure mode was basically the same as that of the J1 joint, since the bolt hole on the T2 joint beam was the same as that of the J1 joint. However, since this joint was a tube connection, the tearing damage of the upper flange of GFRP beam was slighter than that of the J1 joint under the same load.

While for T3 joint, as shown in Fig 6(C), under imposed by bending moment, the failure mode of joint with slotted-hole bolts changed from the failure of bolt hole to shear failure of GFRP beam. Slotted-hole bolts turned into the sliding phase when the displacement reached 20 mm, and bolts gradually slipped into the edge of the bolt hole (the bolt hole was slotted hole). Eventually, bolts contacted completed with the slotted-hole wall of top and bottom flange plate for GFRP beam and bolt-hole wall on the tube. Pressure transmission load between bolt and slotted hole increased gradually, and the proportion of load transferred by friction between bolts and tube components decreased. With the increased in deformation, the main failure mode of this joint was shear failure of GFRP beam under action of shear stress because of elastic modulus of GFRP material was low and easy deformed.

From Figs 5 and 6, it is found that the failure modes of two sets of the specimen were local yielding of lower flange of beam end for tube or angle steel joints, this was because top flange and bottom flange of GFRP beam connected to angle steel or tube by bolts, and withstood tension from the connector (angle steel or tube), to balance external moment and shear force, led to the damage of top and bottom flange of GFRP beam (especially four corners of box cross section).

### Monotonic loading test results and analysis

#### Load-displacement curves

**Fig 7:**
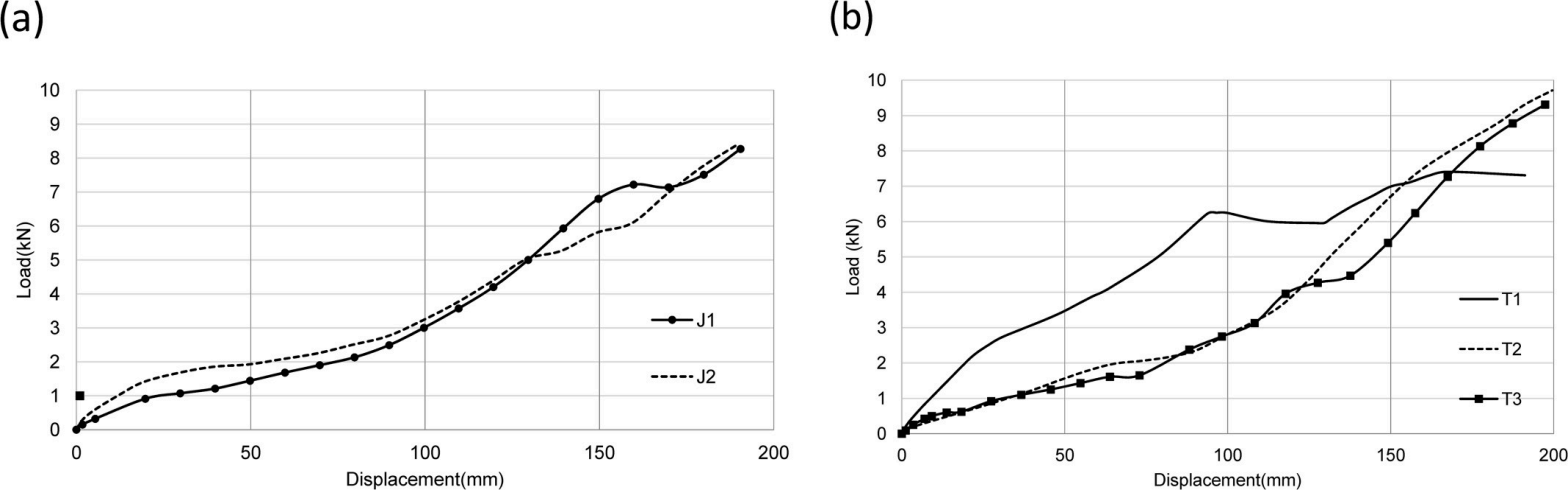
Load-displacement curves. (a) J group;(b) T group.

The load-displacement curves of two groups of joints under monotonic loading were shown in Fig 7. Since the pre-tightening force of bolts for the other four joints did not exceed 10 kN except the T1 joint, it can be seen from figures that load-displacement curves of J1, J2, T2, and T3 joints were similar.

For joint J1, when loading of beam end reached to 1kN, sliding of bolts occurred due to the gap between bolts and bolt holes. At the beginning of the sliding stage, the curve had a lower slope, indicating that the curve had a lower rotational stiffness. For bolted connections, there was friction between connecting members. When the internal force between connecting members was less than external force, the stiffness of the connection would decrease, and sliding friction would occur. Bolt sliding ends when loading of beam end reaches to 1.2 kN.

Unlike joint J1, for J2, the bolt hole on the beam was slotted holes. When loading of beam end increased to 1.1 kN, bolt sliding occurred. Response of bolt connection mainly depended on the frictional sliding mechanism between connecting components (such as bolts, washers, and connectors). Due to the pre-tightening force of bolts, there was friction between connecting parts. When the external force exceeded the internal friction force, bolts began sliding. Bolt sliding ended when beam end load reached 2 kN. At this moment, stiffness of the joint returned to the same as that of joint J1.

However, bolt sliding only occurred when the pre-tightening force of the bolt at joint T1 turned to 15 kN because there were 8 through bolts on the beam when the beam end load was 3 kN (equivalent to a bending moment of 2.85 kN·m at joint). The through bolts shorten the length of the sliding segment of the curve.

For joint T2, the bolt contacted the inner wall of the bolt hole when the beam end load was 0.7 kN. While for T3 joint, when the beam end load reached 0.8 kN, the bolt began to slip in the slotted hole. Because the double-head bolt was adopted, its "sliding" length greater than that of joint T1. When the beam end load increased to 2 kN, the bolt stopped sliding.

It can be seen from the load-displacement curves of joints in Fig 7(A) that the ultimate bearing capacity of both J1 and J2 was 7.35 kN, indicating that the slotted holes method would not increase the bearing capacity of joints.

#### Moment-rotation curves

**Fig 8:**
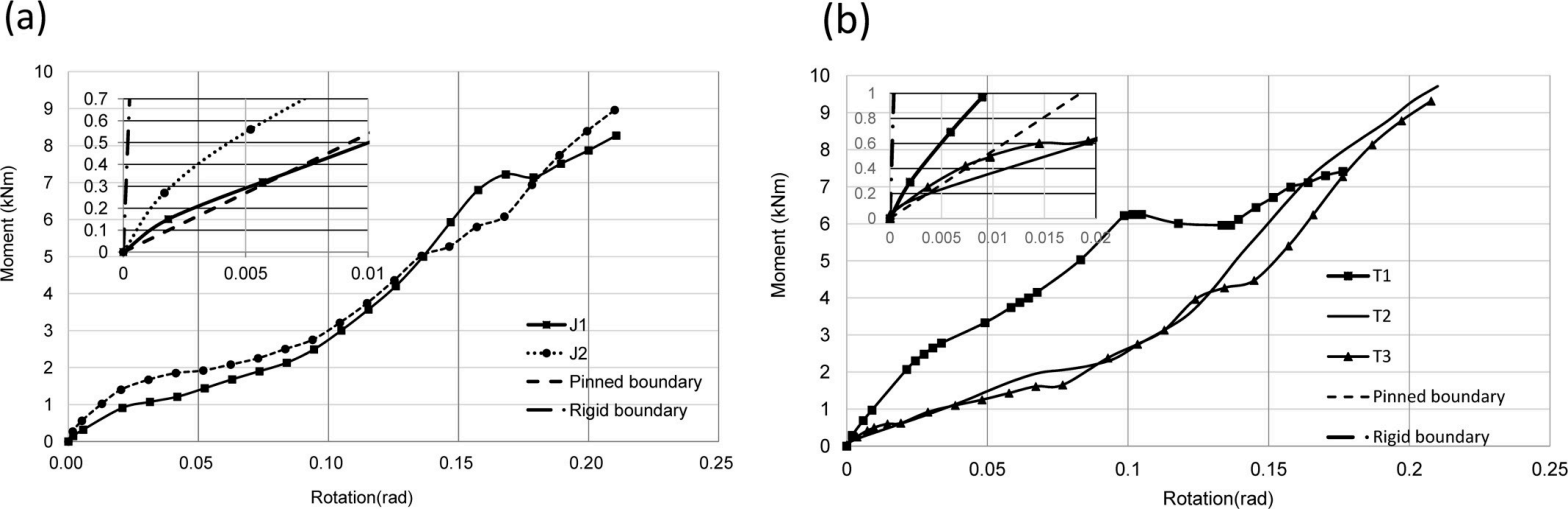
Moment-rotation curves. (a) J group;(b) T group.

The bending moment-rotation curves of joints are shown in Fig 8. It can be seen that the bending moment gradually increased with rotation angles. Stiffness of joints decreased as bolts on GFRP beam broken and beam flange plate torn. For these two group joints, the variation tendency for these bending moment vs rotation curves was almost the same; the main reason was failure modes of these two groups of joints were the same.

For joints in group *J*, the ultimate bending moment of J1 and J2 was 7.1 kN·m, and initial rotational stiffness values were almost the same (see Table 3). It can be obtained that the way of slotted holes will not increase the ultimate bearing capacity of joints. Because J2 adopted the way of slotted holes, bolt slippage occurs when the bending moment of the joint was 1 kN·m, and external force exceeded internal friction force, leading to a reduction of stiffness of joints. When the bending moment of joints reached 1.8 kN·m, bolt slippage stopped. Compared with joint J1, the bolt "slippage" section of joint J2 was longer.

**Table 3:** Plastic rotation, initial rotational stiffness and yield stiffness for specimens.

Specimens	Plastic rotation *θ*_*P*_ (rad)	Initial rotational stiffness *K*_*i*_ (kN·m/rad)	Yield stiffness *K*_*y*_ (kN·m/rad)
J1	0.190	85	40.53
J2	0.225	98	31.61
T1	0.116	145	52.62
T2	0.21	83	45.47
T3	0.259	75	34.11

While for the T joints, bolt pre-tightening force of 15 kN was imposed to T1, and eight go-through bolts adopted to connect the GFRP beam and sleeve connector, initial rotational stiffness became bigger (see Table 3). When the bending moment of T3 was 0.7 kN·m, the bolt began to slip. Since this joint uses single side bolts, the failure mode of the joint was local buckling among bolts and the lower flange of GFRP beam. The bolt preloading force of joint T2 less than that of joint J1, but the initial rotational stiffness of J1 and T2 was roughly the same, so it can be concluded that the bending moment bearing capacity of the tube connector was greater than that of angle steel connector.

According to European Code [36], the beam-column connection was classified by the initial rotational stiffness of the connection. Initial rotational stiffness of this kind of joints was greater than 54 kN·m/rad, and less than 2714 kN·m/rad, which was close to the boundary of semi-rigid joints. These two boundary lines were also drawn in Fig 8. It can be seen from Fig 8 and Table 3 that these two groups of joints were semi-rigid joints. For J1, J2, T2, and T3, the initial rotational stiffness values were closer to the hinge point boundary.

#### Load-stress analysis

**Fig 9:**
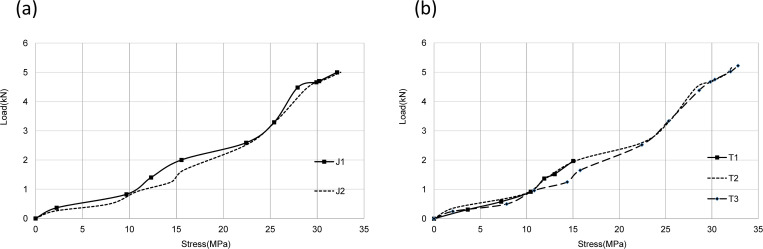
Load-stress curves. (a) J group;(b) T group.

According to strain gauge data of SG3 at the web of GFRP beam, a load-stress curve could be obtained as shown in Fig 9. Since no side plates were added to the web of column for the T1 joint to reinforce the joint, the column of the T1 specimen damaged with a maximum strain of SG3 of 762 με, equivalent to a maximum stress of 22.2 MPa, which did not reach the ultimate shear stress of GFRP beam yet. The other four GFRP beams reached the ultimate shear stress before yield failure. Except for T1, failure modes of the four specimens were tearing failure at the junction of beam flange and web, which mainly concentrated in the four corners of the tubular section. The beam-column connection areas of J1, J2, T2, and T3 joints were reinforced, no damage occurred to the GFRP column during the tests.

#### Plastic rotation

The peak of bending moment corresponding to the moment-rotation curve was defined as the ultimate bending moment, ***M***_***p***_, and corresponding rotation was ***θ***_***p***_.Plastic rotation could be used to evaluate the plastic rotation capacity of joints under loading. Plastic rotation ***θ***_***P***_ was defined as plastic displacement of the beam end divided by the calculated length of the beam. For joints in this paper, it was calculated according to Eq (3)

$$\theta_{P}=(\delta_{CL2}-\delta_{CL1})/L_{CL1,CL2}$$ (3)

Where *δ*_*CL*1_ and *δ*_*CL*2_ are were vertical displacements of the lower flange of the beam at points *CL*1 and *CL*2, respectively, and *L*_*CL*1,*CL*2_ was the length between points *CL*1 and *CL*2.

Table 3 showed the plastic rotation of each specimen calculated according to Eq (3). The plastic rotation of both groups of joints was greater than 0.05 rad, indicating both groups had good plastic rotation ability. The plastic rotation of the J2 specimen, was 1.18 times that of the J1 specimen, indicating that the plastic rotation ability of joints would be increased by way of slotted holes. The average plastic rotation of tube connector specimen was 1.1 times that of angle steel specimen, which indicates tube connector specimen had a better plastic rotation ability than angle steel joints.

#### Initial rotational stiffness and yield stiffness

The initial rotational stiffness is defined as the tangent slope of the curves of moment-rotation at the linear stage [37], which is shown in Table 3. Comparison of initial stiffness of two groups of joints, the average initial rotational stiffness of specimens in Group *T* was 1.1 times that of specimens in Group *J*, which indicates tube connector specimens had a superior flexural capacity of joints and larger space of building.

**Fig 10:**
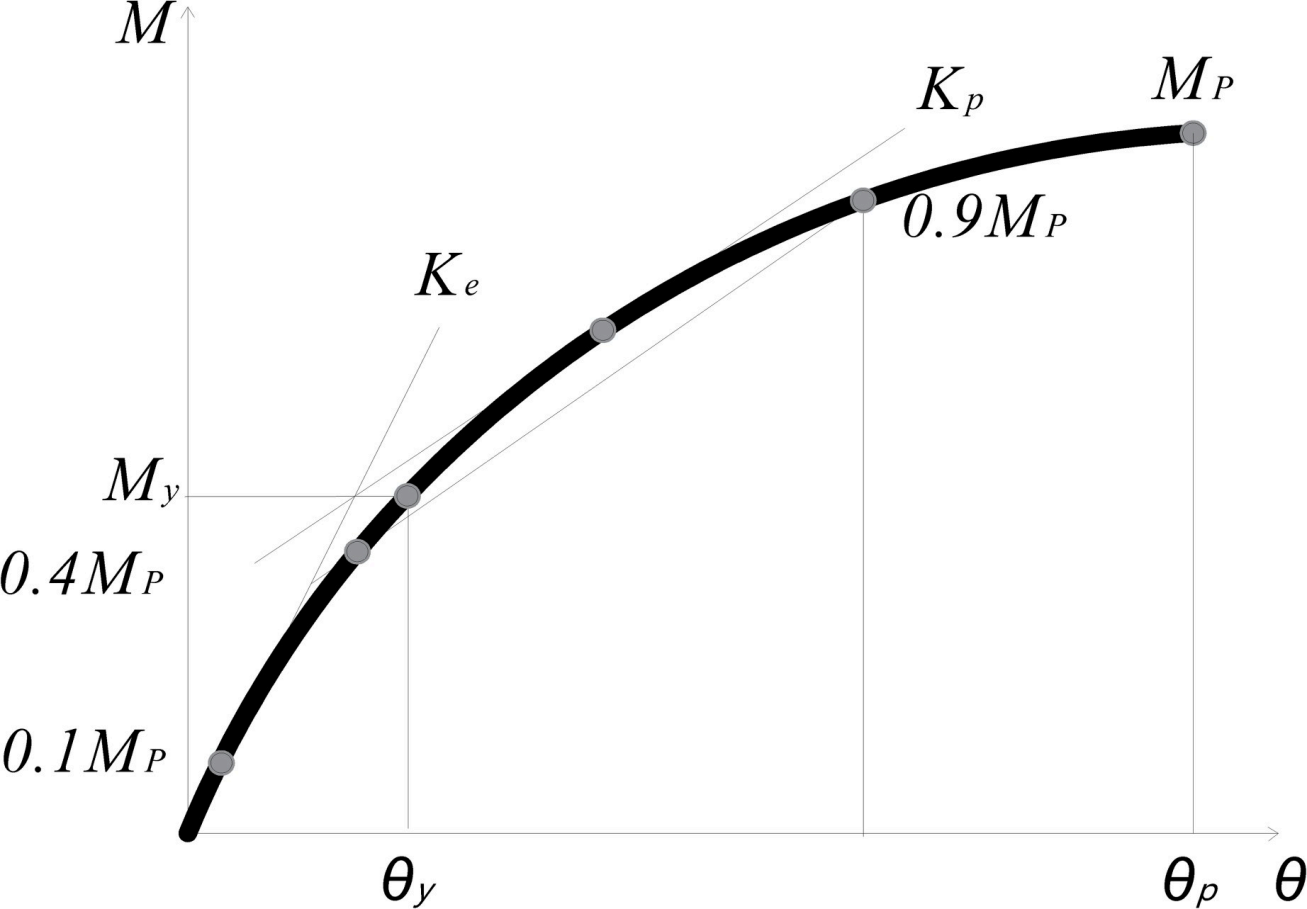
Principle diagram of Y&K method.

The principle of Y&K (Fig 10) method [38,39] was adopted to evaluate the yield stiffness of these two groups of joints. The bending moment and rotation corresponding to the yield point were *M*_*y*_ and *θ*_*y*_ respectively. For bolted joints connected by angle steel or tube, the rotation at the end of bolt sliding can be regarded as yield rotation, and the corresponding bending moment was yield moment, *M*_*y*_. The yield stiffness *K*_*y*_ was calculated according to Eq (4) [38,39].


$$K_{y}=\frac{M_{y}}{\theta_{y}}$$ (4)

The calculated yield stiffness *K*_*y*_ of joints is shown in Table 3. From this table, the yield stiffness of specimens in Group *T* was also greater than that of specimens in Group *J*, and the average stiffness of tube connector joints was 1.22 times that of angle steel joints, indicating that mechanical properties of tube joints were better than those of angle steel joints.

#### Influence of side plates on GFRP beam-column joints

Failure modes of J1, J2, T2 and T3 specimens with side plates added was tearing failure of beam flange, while failure mode of T1 specimens without side plates was that column was crushed by end plates. As shown in Fig 8, the ultimate bending moment capacity for T2 and T3 joints was greater than that of the T1 joint (ultimate bending moment capacity increased by 30%). To avoid premature damage of joint like separation of column flange from web, reinforcement was needed to connecting area of beam-column joints. It was suggested that side plate of the column could effectively increase shear capacity of the joint domain, and meet the strong column weak beam design principle.

#### Effect of pre-tightening force for bolts on GFRP beam-column joints

By comparing the bending moment-rotation curves of T group joints in Fig 8(B), it could be concluded that bolt preload had the following effects on beam-column joints: the greater the bolt preload is, the greater the sliding load of joints and initial stiffness of joints are. However, excessive preload should not be allowed because the elastic modulus of GFRP material was lower than steel, and much bolt preload would crush the GFRP beam. Therefore, the proposal range of preload could be between 10 kN and 15 kN.

In general, it is easy to see that bolting connections with slotted holes are helpful to improve the seismic capacity and rotational capacity of the joints, preventing joints from being damaged in rare earthquakes. However, too large rotation angle has the negative impact on the deformation and bearing capacity of the whole structure. Therefore, in practical design, a suitable dimension of slotted holes can be determined by calculation, which can not only ensure the greater energy dissipation capacity of the joints, but also ensure that the deformation and bearing capacity of the whole structure meet the standard requirements.

## Conclusions

In this paper, a new connection method (slotted-hole bolts connection) was proposed and exmained based on the traditional angle steel and tube connectors to GFRP beam-column joints. Two groups of joints (angle steel joints and tube joints) were experimentally investigated in this study. The test results were obtained by monotonic loading test, including failure mode of specimens, load-displacement curves, bending moment-rotation curves, load-stress curves, an initial rotational stiffness and plastic rotation. A comprehensive comparison was conducted on the four parameters, including different connectors (angle steel and tube), types of bolt holes (slotted holes and ordinary round holes), GFRP column with or without side plate, and different bolt pre-tightening force. The following conclusions could be drawn.

1. For all bolted connections, separation and failure of the lower flange of the GFRP beam web were observed. The separation of the web from the flange was caused by the compression of the bolt on the upper flange of the GFRP beam and the tension of the lower flange of the connector. When joints were damaged, the end plates deformed significantly.

2. Average plastic rotation of tube connector joints was 1.1 times that of angle steel joints, and the average plastic rotation of slotted-hole joints was 1.18 times that of ordinary round joints. The "sliding" section of load-displacement curves of slotted-hole joints was more obvious than that of ordinary round joints.

3. According to strain gauge values from the test, the failure mode of the T1 joint without side plate was the GFRP column crush caused by the end plates, while there was no failure on the GFRP column of other joints with side plates. After reinforcing on the beam-column connection area with side plates, the bending moment bearing capacity increased by 30%.

4. The increase in the bolt pre-tightening force will lead to a higher friction between the plates. Meanwhile, the increase in the bolt slip load will lead to a higher initial rotational stiffness.

## Supporting information

S1 Appendix (DOCX)

S1 File Load-displacement curves of Group J. (XLSX)

S2 File Load-displacement curves of Group T. (XLSX)

S3 File Moment-rotation curves of Group J. (XLSX)

S4 File Moment-rotation curves of Group T. (XLSX)

S5 File Load-stress curves of Group J. (XLSX)

S6 File Load-stress curves of Group T. (XLSX)
