# Supplementary material for: Monotonic loading performance of GFRP beam-column joints connected with slotted–hole bolts
Source: PLoS One. 2022 Jul 27;17(7):e0272136. doi: 10.1371/journal.pone.0272136 (PMC9328552; doi:10.1371/journal.pone.0272136)
Supplement: S1 Appendix — (DOCX) [file pone.0272136.s001.docx]

**Supporting Information**

**S1 Appendix**

**Appendix A: Plastic rotation, Initial rotational stiffness and Yield stiffness**

**(1) Plastic rotation**

Plastic rotation $\theta_{P}$ was defined as plastic displacement of the beam end divided by the calculated length of the beam. For joints in this paper, it was calculated according to Eq (S1)

$\theta_{P}$= ($\delta_{CL2}$－$\delta_{CL1}$) /$L_{CL1,CL2}$ (S1)

Where$\delta_{CL1}$ and $\delta_{CL2}$were vertical displacements of the lower flange of the beam at points *CL*1 and *CL*2, respectively, measured by a displacement meter mounted at the location where from the end of beam 300mm and 650 mm, respectively , as shown in S1 Fig, and$L_{CL1,CL2}$ was the length between points *CL*1 and *CL*2, 650 mm.S1 Table showed the plastic rotation of each specimen calculated according to Eq (S1).

**
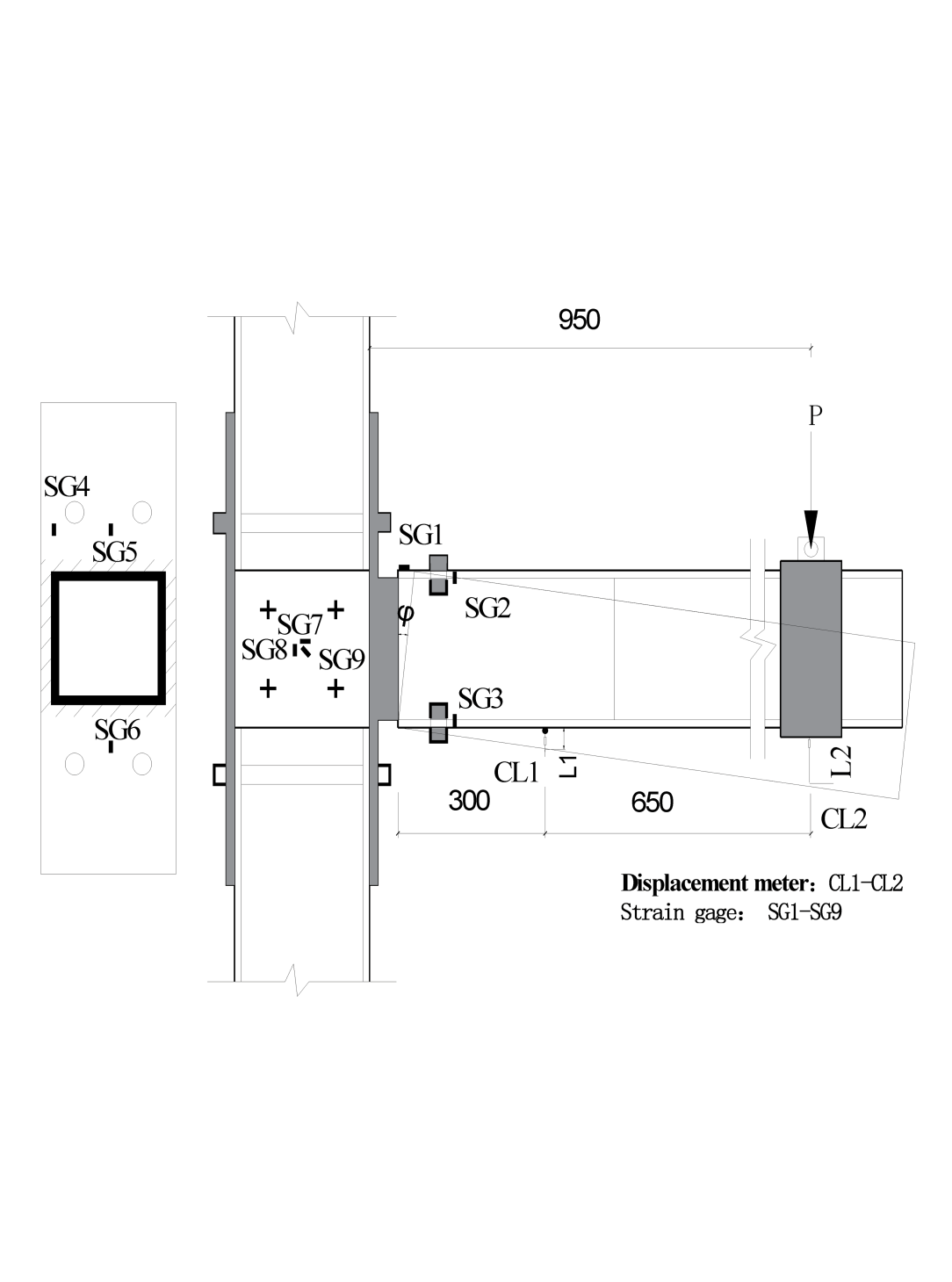
**

**S1 Fig. Diagram of Strain gage and displacement meter.**

**S1 Table.** **Plastic rotation, Initial rotational stiffness and Yield stiffness for specimens**

| Specimens | Plastic rotation $\theta_{P}$（rad） | Initial rotational stiffness $K_{i}$ (kN·m/rad) | Yield stiffness$K_{y}$（kN·m/rad） |
| --- | --- | --- | --- |
| J1 | 0.190 | 85 | 40.53 |
| J2 | 0.225 | 98 | 31.61 |
| T1 | 0.116 | 145 | 52.62 |
| T2 | 0.21 | 83 | 45.47 |
| T3 | 0.259 | 75 | 34.11 |

**(2) Initial rotational stiffness**

The initial rotational stiffness is defined as the tangent slope of the curves of moment-rotation at the linear stage, which is shown in S1 Table. This is the method referenced in Ref. [S1**]**.The curves of moment-rotation for determining the initial rotational stiffness come from S2 Fig, which obtained either by recorded data by 50T electro-hydraulic servo static loading system( S3 Fig**)** or measured by displacement meters(S1 Fig).

**
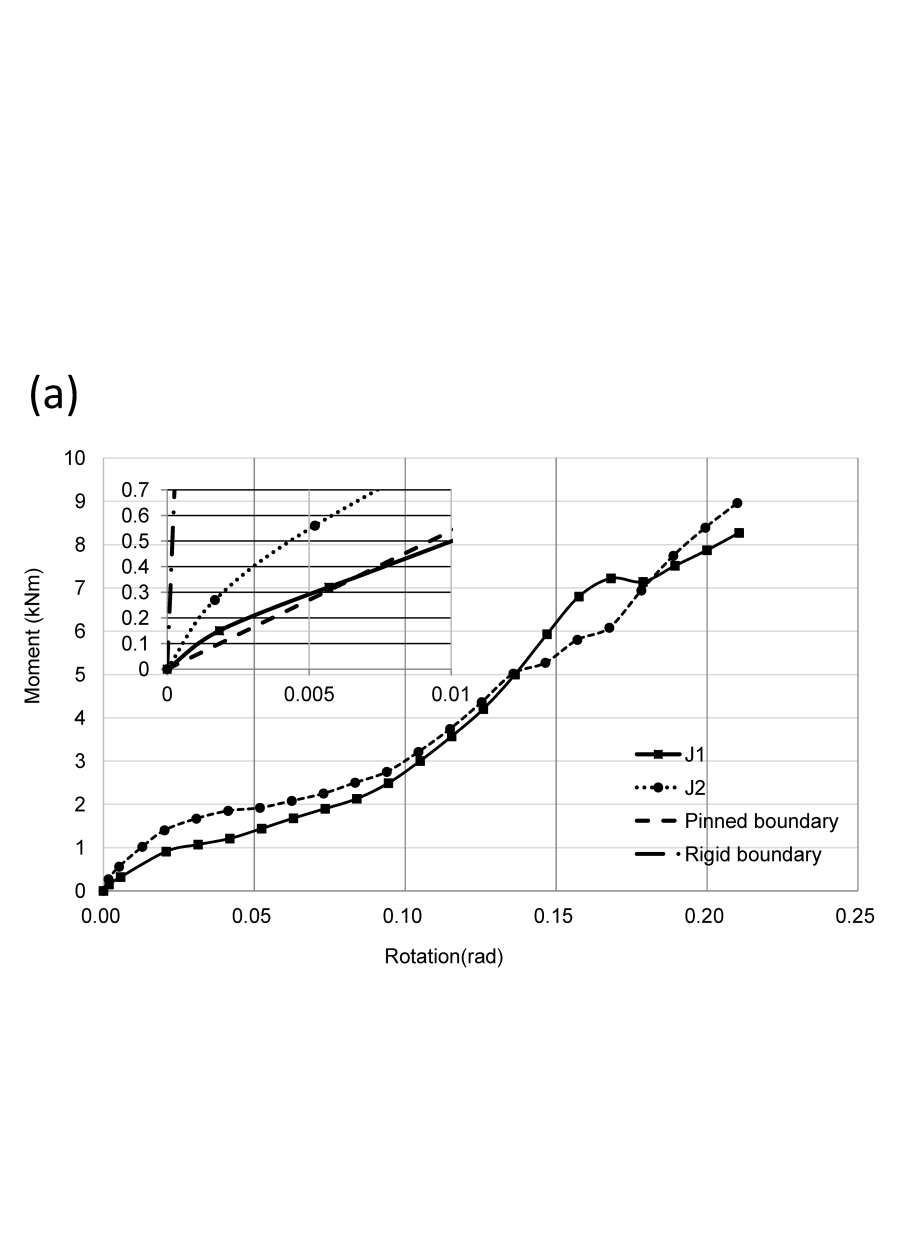
**

**
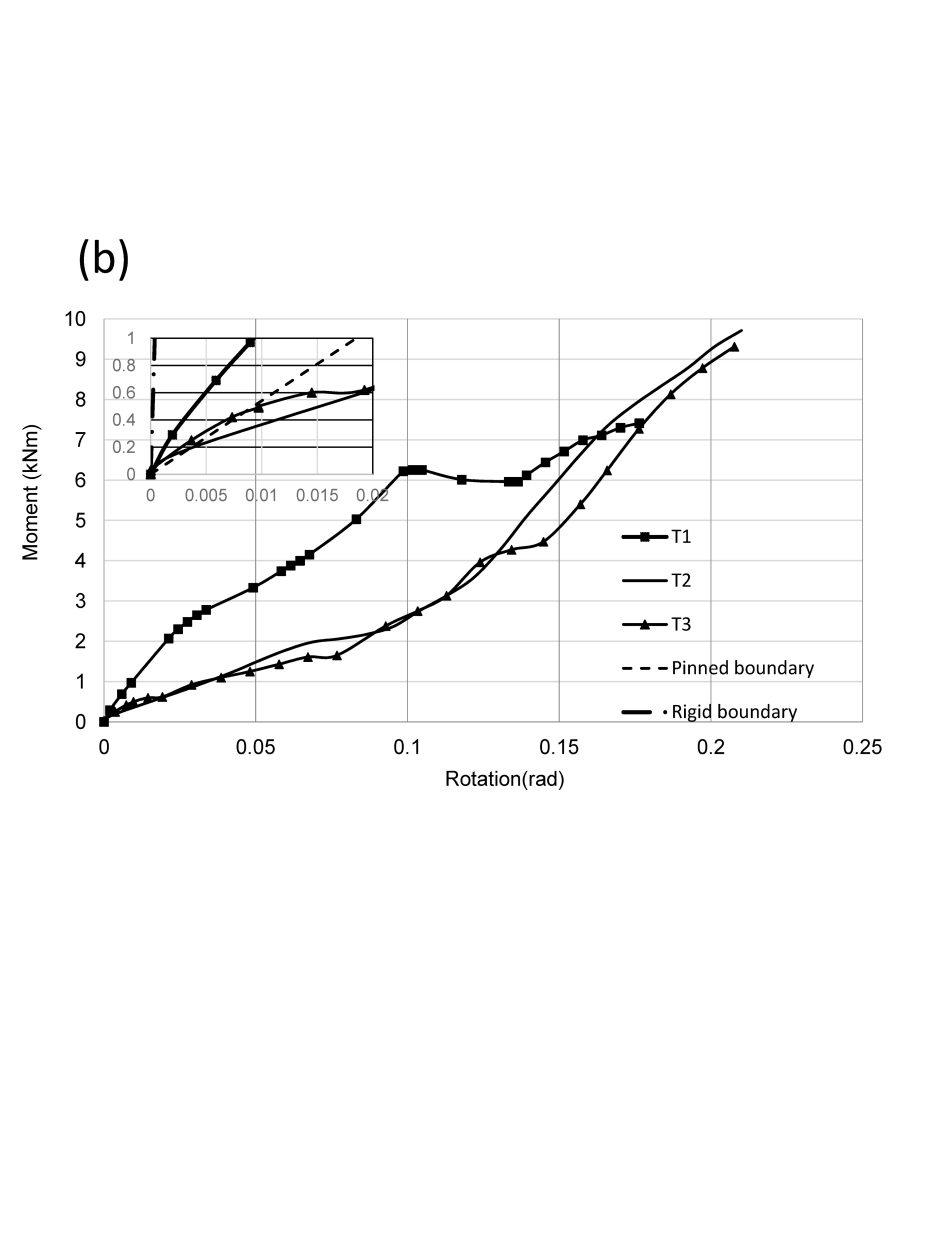
**

**S2 Fig. Moment-rotation curves** :(a) J group;(b) T group.

**
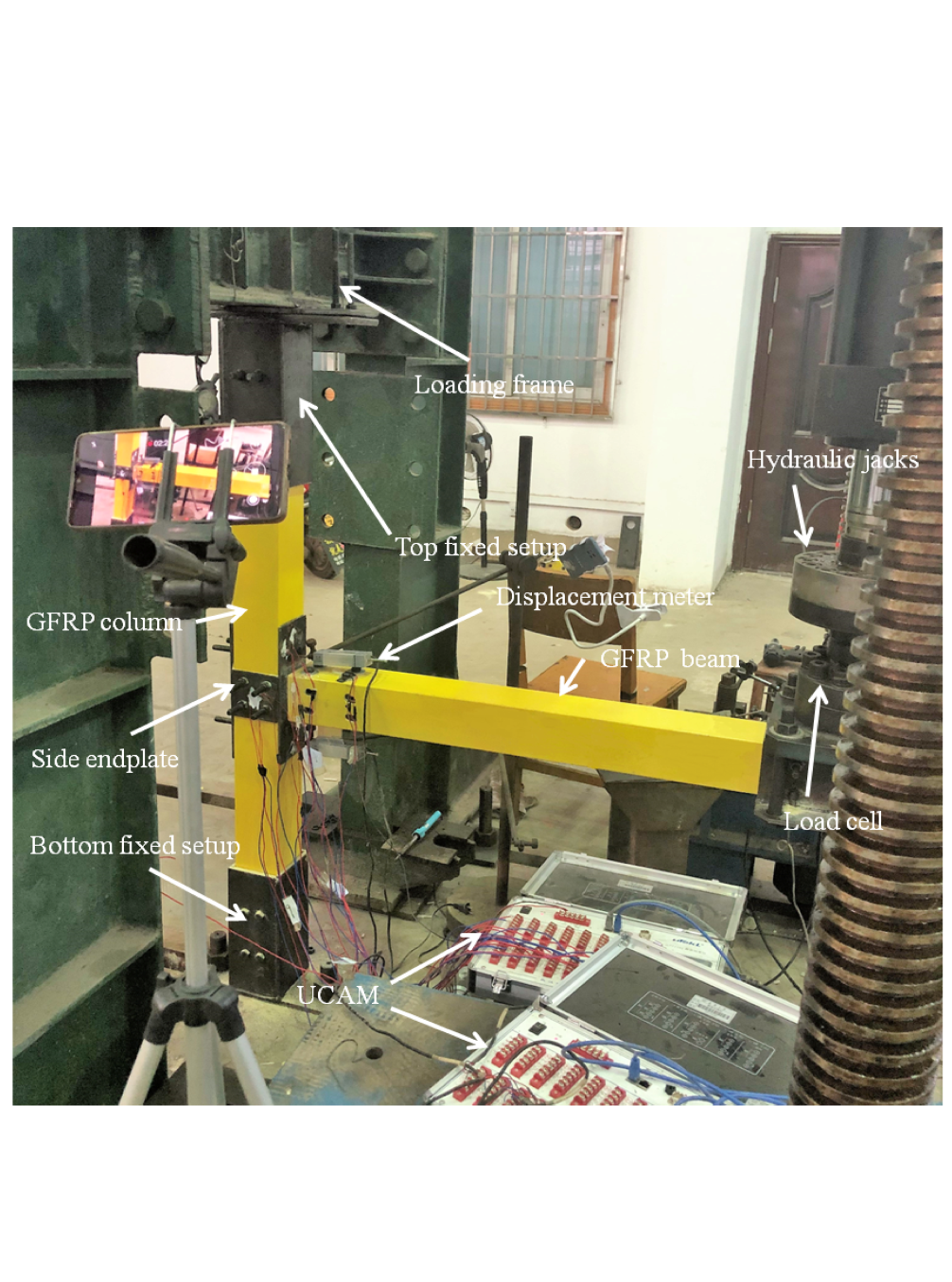
**

**S3 Fig. Experimental set-up for specimens.**

**(3) Yield stiffness**

The principle of Y&K (S4 Fig) method[S2-S3] was adopted to evaluate the yield stiffness of these two groups of joints in this paper. The bending moment and rotation corresponding to the yield point were $M_{y}$ and $\theta_{y}$respectively. For bolted joints connected by angle steel or tube, the rotation at the end of bolt sliding can be regarded as yield rotation, $\theta_{y} ,$and the corresponding bending moment was yield moment, $M_{y}$[S2-S3]. As a result, the yield stiffness $K_{y}$was calculated according to Eq (S2)

$K_{y}=\frac{M_{y}}{\theta_{y}} \mathbf{(}S2\mathbf{)}$

The calculated yield stiffness$K_{y}$ of joints is shown in S1 Table.

**
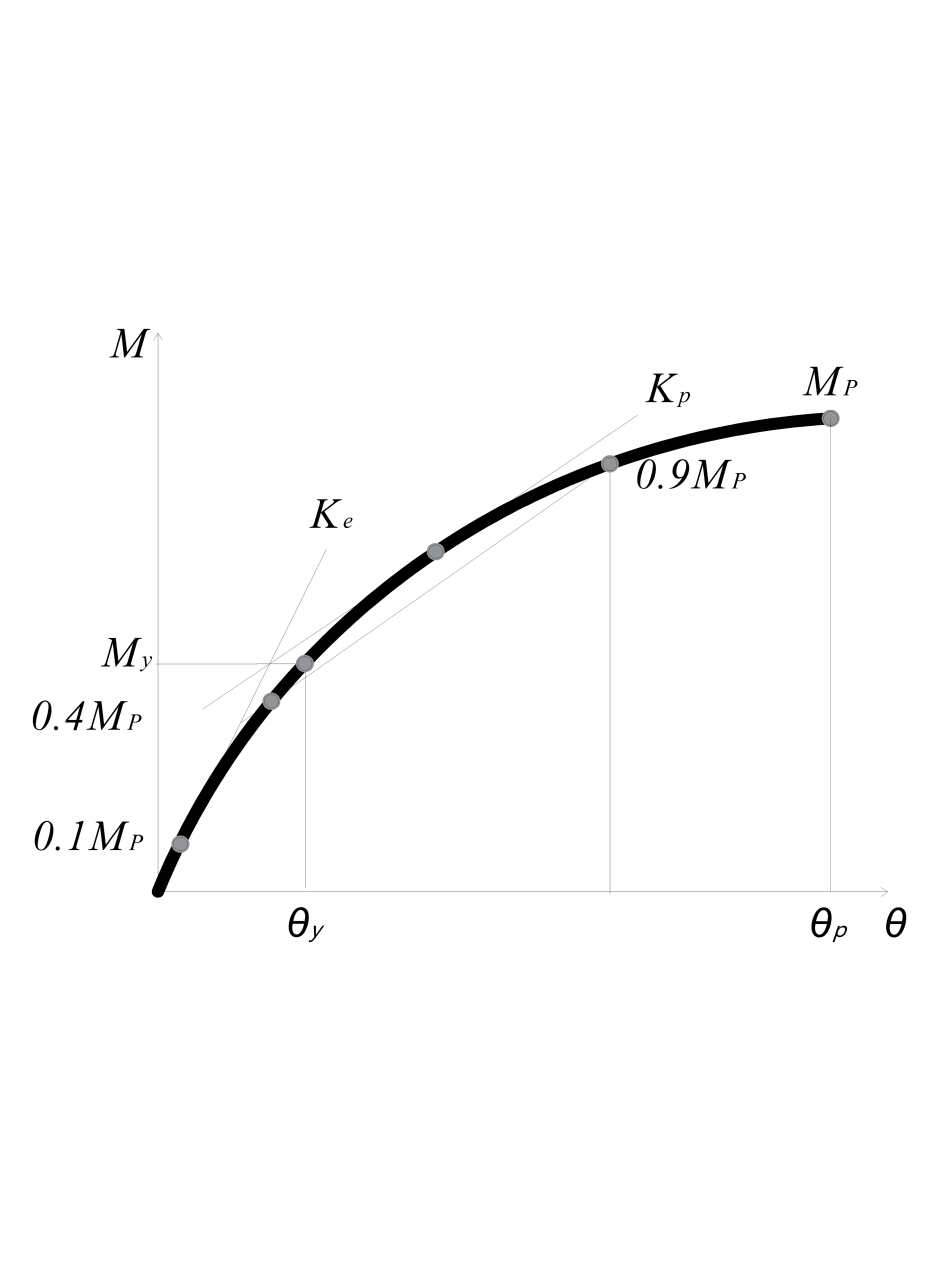
**

**S4 Fig. Principle diagram of Y&K method**.

**Appendix B: Load-stress analysis**

The Load-stress curves were used instead of the stress-strain analysis in this paper. The benefit to show load-stress is that in order to obtain the real-time relationship between the bearing capacity of the beam end and the material stress, we can intuitively know the ultimate bearing capacity of the beam when the material damaged. The load-stress data from experiments can be found in S6 and S7 Files.

**Appendix C: Influence of side plates**

To avoid premature damage of joint like separation of column flange from web, side plates were needed to add the stiffness of connecting area of beam-column joints. Experimental results indicated that failure modes of T2, T3, J1, and J2 specimens with side plates was tearing failure of beam flange, while failure mode of T1 specimens without side plates was that column was crushed by end plates, which is shown in S5 and S6 Figs.

**
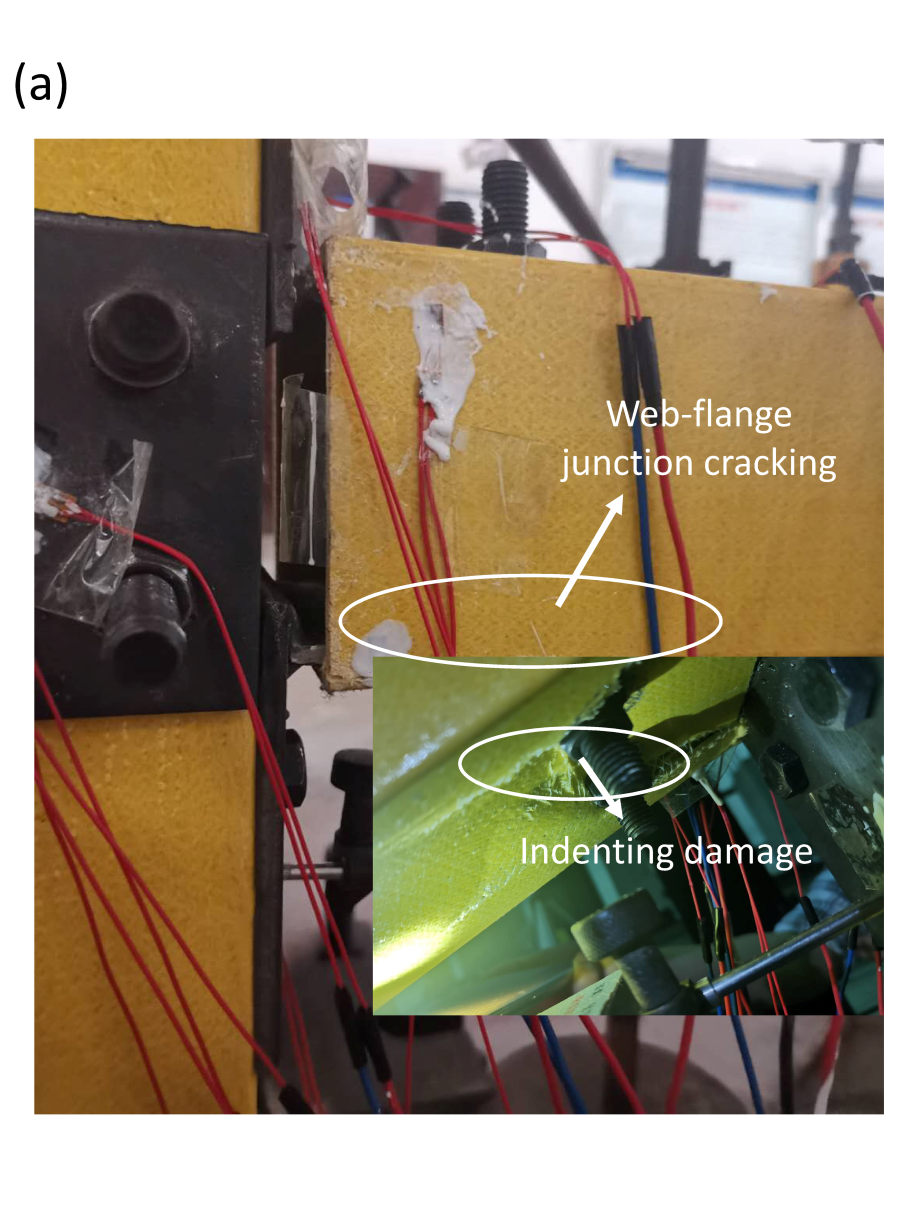
**
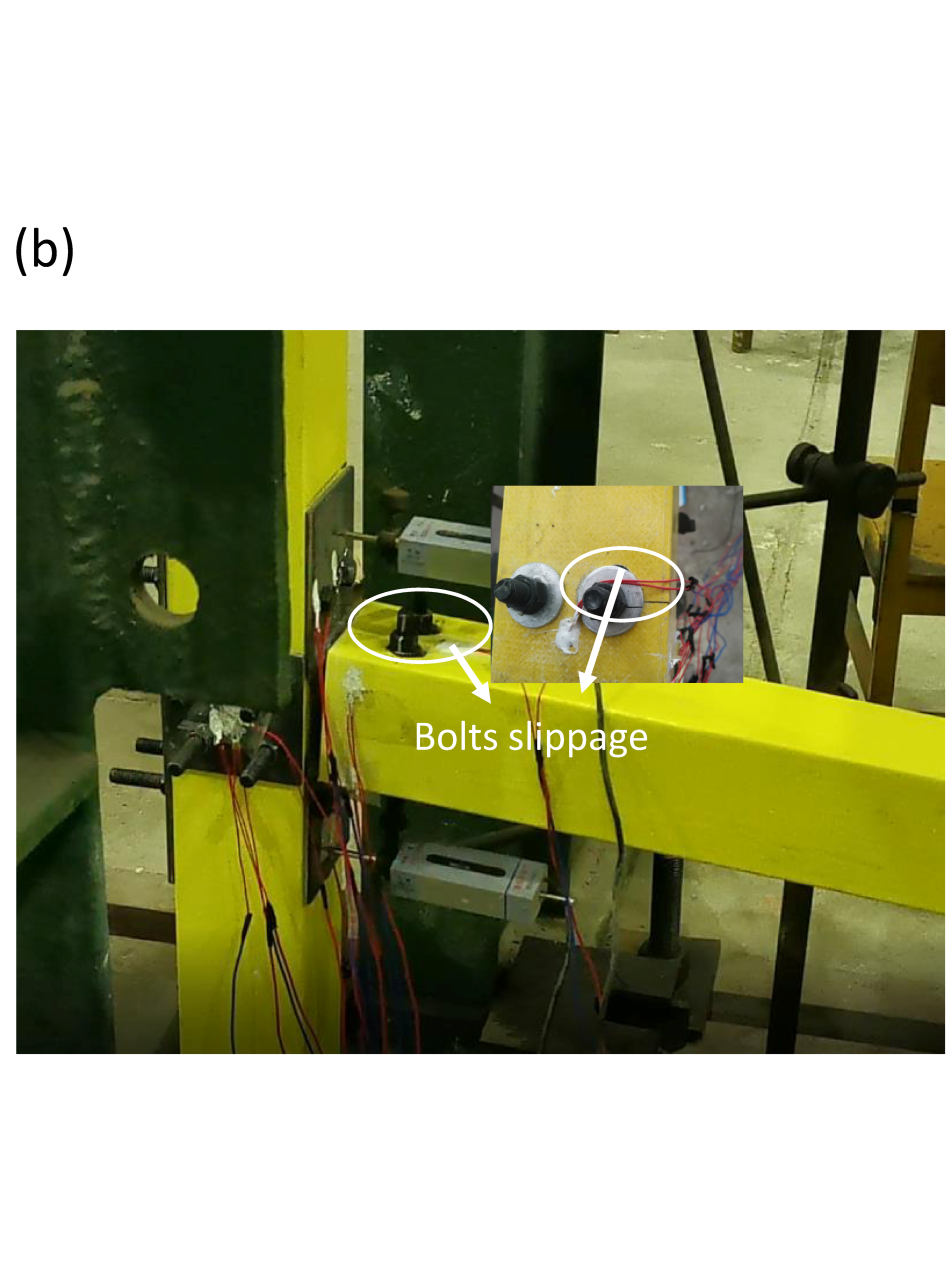


**S5 Fig. Failure modes of specimens of group J**:(a) J1; (b) J2.

**
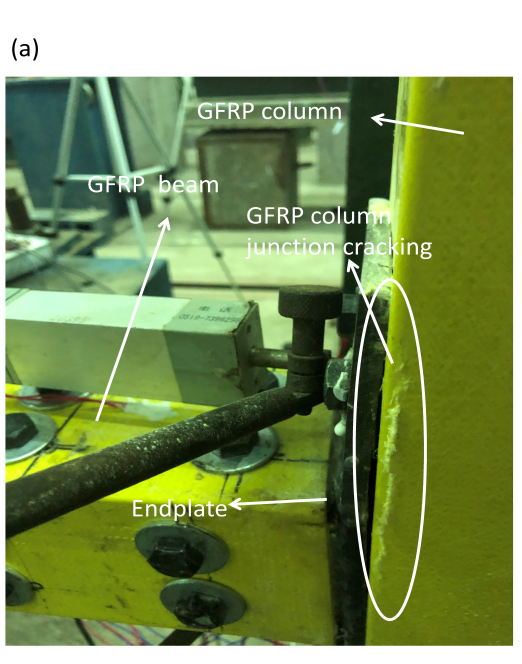

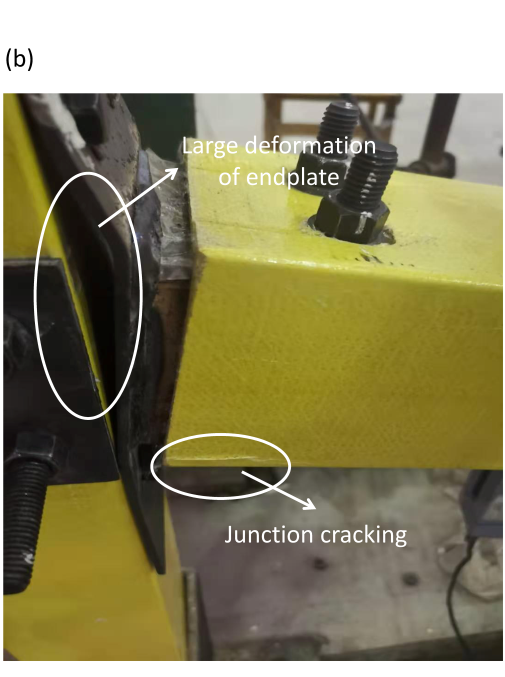
**

**
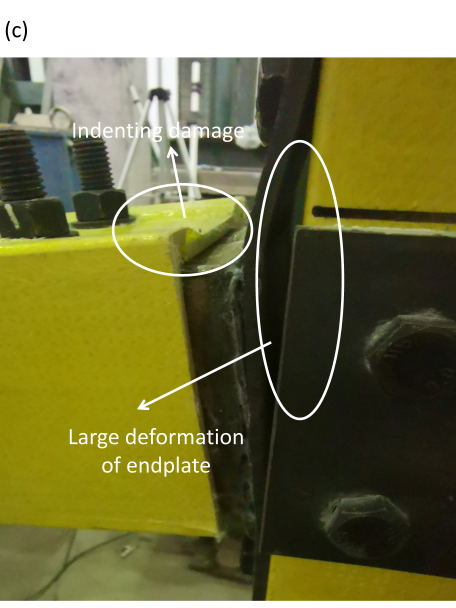
**

**S6 Fig. Failure modes of specimens of group T**:(a) T1; (b) T2; (c) T3.

It was also found from S2 and S7 Figs that the ultimate bending moment capacity for T2 and T3 joints was greater than that of the T1 joint (ultimate bending moment capacity increased by 30%). This is due to there was no obvious yield point in the bending moment-rotation curves of T2 and T3 specimens while T1 specimen has a yield point, as is shown in the S2 Fig, so the bearing capacity of specimens with side plates was improved.

**
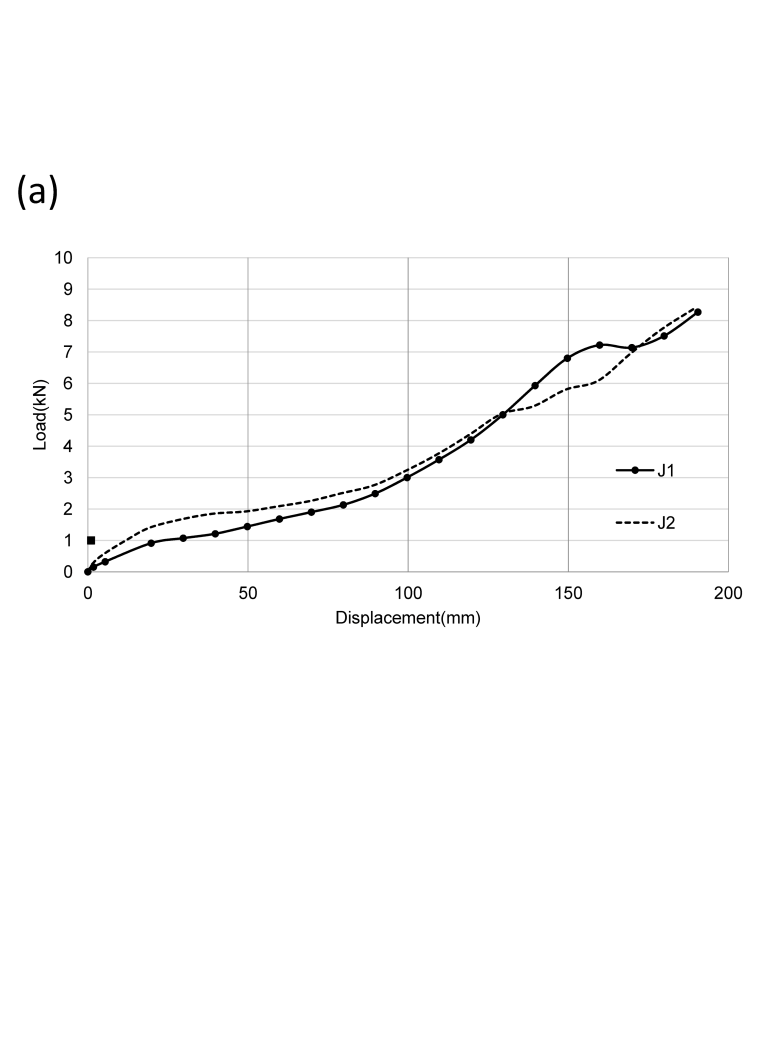
**

**
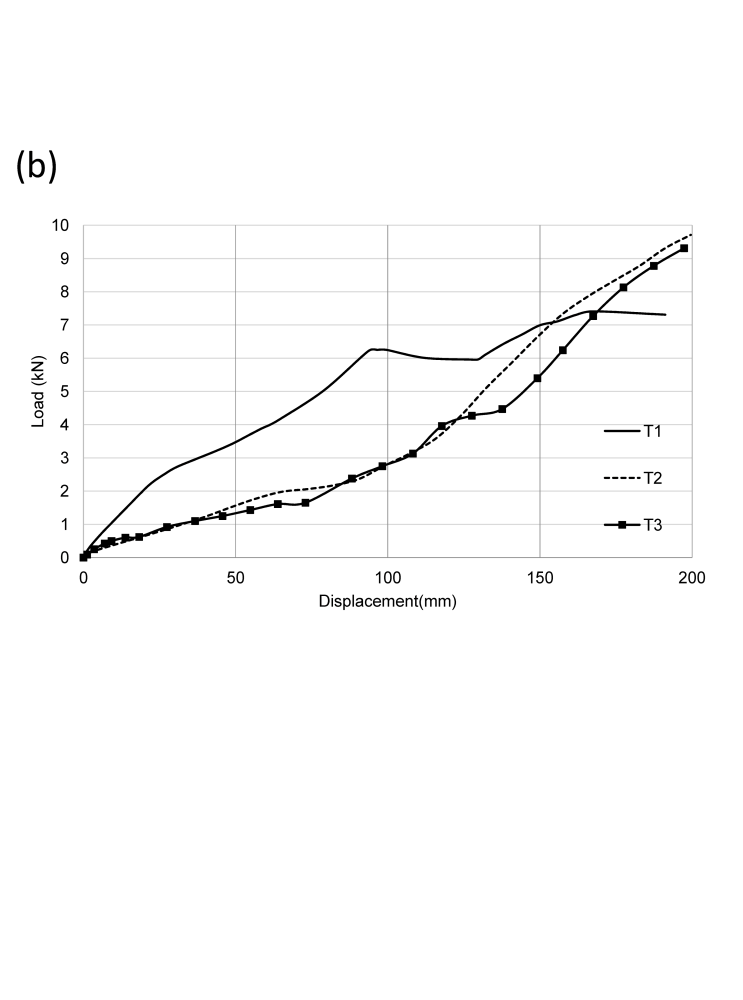
**

**S7 Fig. Load-displacement curves** :(a) J group;(b) T group.

# References

S1. Zhang ZJ ,Bai Y, Xiao X. Bonded sleeve connections for joining tubular glass fiber-reinforced polymer beams and columns: experimental and numerical studies, Journal of Composites for Construction, 2018, 22(4): 04018019.doi: 10.1061/(ASCE)CC.1943-5614.000085 3.

S2. Yasumura M, Kawai N. Estimating seismic performance of wood-framed structure// Proceeding of 1998 I.W.E.C. Swizerland,1998:564-571.

S3. Zhao Y, He MJ, Ma RL.Rotational performance of pre-stressed multibolted glulam connection with square tubes. Journal of Tongji university (natural science), 2016,44(8):1182-1189. doi:10.11908/j.issn.0253-374x,2016.08.007. (in Chinese).
